# Net renal perfusion - an emerging concept during development of AKI in the critically ill

**DOI:** 10.1080/0886022X.2026.2633845

**Published:** 2026-03-02

**Authors:** Csaba Kopitkó, Zsanett Tóth

**Affiliations:** a Department of Anesthesiology and Intensive Care Therapy, Hospital of Defence Forces, Budapest, Budapest, Hungary; b Doctoral College, Semmelweis University, Budapest, Hungary

## Introduction

In healthy individuals, kidneys reliably maintain adequate renal perfusion pressure (RPP) for ultrafiltration and ensure sufficient oxygen supply to renal tissue through autoregulation. On the other end of the clinical spectrum, the autoregulation deteriorates not only due to changes in microcirculation, but also due to microcirculatory disturbances in critically ill patients. Therefore, it is essential to monitor pulse wave velocity in renal arteries and veins alongside all the measurable parameters such as mean arterial pressure (MAP), central venous pressure (CVP), intra-abdominal pressure (IAP), mean perfusion pressure (MPP = MAP-CVP), abdominal perfusion pressure (APP = MAP-IAP). A promising parameter (MAP-IAP-CVP-Pmean, where ‘Pmean’ stands for mean airway pressure) was described in both non-cardiac and cardiac postoperative patients [1,2].

This ‘new’ concept requires a brief explanation. The cardiology literature uses MAP-CVP, whereas the surgical literature uses MAP-IAP for estimating RPP. Although CVP and IAP are of the same order of magnitude, authors still adhere to the formula commonly used in their respective fields. This may be partly because the main contributing factor is CVP elevation in cardiology patients and IAP elevation in surgical patients. However, this concept is not entirely new. Until 2016, the WSACS guidelines on increased IAP included the filtration gradient (FG = MAP−2 × IAP) [3,4]. This is because higher IAP impedes glomerular filtration by transmitting in both the venous and urinary systems. Pmean primarily leads to an increase in CVP and lymphatic system pressure; however, the latter has not been considered in previous studies. Due to the tight fibrous capsules of the kidneys, an exponential increase in pressure occurs when drainage is obstructed and fluid remains inside, therefore all decompression-related factors must be taken into consideration [1]. Dang et al. have confirmed the usefulness of the recommended formula for cardiac surgery patients. For this patient group, the perioperative increase in IAP is a well-known but under-researched phenomenon [2].

Research definitively shows a tendency that partial oxygen pressure measured in urine during the perioperative period is a suitable biomarker for the development of postoperative acute renal failure. This value indicates oxygen deficiency in the kidneys due to low oxygen pressure in the urine [5,6].

While the role of circulatory changes in the development of several types of AKI is generally accepted, these theories are mainly based on experimental studies. In a detailed review paper, Trask-Marino et al. discuss the renal haemodynamics observed in patients with preserved and reduced ejection fraction, including clinical data [7].

## Contributions to the article collection

The 5 manuscripts included in this collection reflect the breadth of postoperative acute kidney injury (AKI) and AKI of cardiac or septic origin.

### Critical MAP

MAP is a continuously and real-time measurable hemodynamic parameter that provides important information about the blood supply and oxygenation of organs and tissues. Monitoring of MAP can provide insight into renal blood flow (RBF) and the risk of AKI in critically ill patients. The commonly accepted safety threshold is to maintain MAP above 65 mmHg.

Wang et al. analyzed the Medical Information Mart for Intensive Care IV (MIMIC-IV) database to explore the role of MAP in the development of acute kidney injury associated with heart failure [8]. The authors identified five groups based on MAP trajectories within the first 24 h. The medium MAP group (85–91 mmHg) exhibited the best, while the high-to-low MAP group demonstrated the least favorable prognosis. In the latter group, the most marked decline in MAP was observed, even though MAP remained over 70 mmHg.

The study by Zhao et al. in which the authors retrieved data from 17,874 patients using the MIMIC-IV and eICU databases, supports the role of individual target MAP [9]. They found that, a MAP of 65–73 mmHg is sufficient for patients without history of hypertension, but MAP of 70–80 mmHg is beneficial for patients with a history of hypertension in case of septic AKI.

In line with this, a retrospective observational study of patients requiring continuous renal replacement therapy (CRRT) found that lower MAP levels were associated with higher 28- and 90-day mortality rates (*p* < 0.00001 for both) [10]. The cutoff value for MAP, as determined by the ROC curve, was 71.85 mmHg. Hao Dong et al. published similar results in patients with acute pancreatitis who required intensive care [11].

### Role of venous congestion

It is supposed that mean perfusion pressure may become a significant parameter in the treatment of critically ill patients in the future. Fang et al. considered not only MAP but also CVP to be important in assessing renal circulation based on the MIMIC-IV database [12]. CVP indicates the blood outflow capacity from the kidneys, so the difference between MAP and CVP values can be used to calculate mean perfusion pressure (MPP), which is a more accurate and reliable marker of blood pressure in the renal vessels than MAP monitoring alone. An increasing CVP indicates an elevation in pressure resulting from venous congestion. This causes an elevation in interstitial pressure in the kidney due to the rigid capsule, resulting in renal tamponade. This impairs capillary circulation and causes acute renal failure. The findings of the present study indicate that, in cases of sepsis-induced acute renal failure, MPP maintained above 60 mmHg is associated with a reduced 90-day mortality rate. Patients with MPP of 40–50 mmHg were older and sicker than those in the other groups. They had a higher prevalence of hypertension and diabetes, as well as worse laboratory parameters. They also had the highest need for vasoactive drugs. As expected, this group had the highest proportion of patients requiring CRRT, and was associated with the highest mortality rates across all categories.

In addition to monitoring tissue perfusion, it is important to maintain and improve renal circulation. Studies have been conducted by Fan et al. on how ß-blockers reduce 28-90-day mortality in sepsis-induced acute renal failure in a manner that is not yet fully understood [13]. Their beneficial effect is probably due to their inhibition of sympathetic tone and reduction of inflammation, thereby helping to maintain microcirculation. Unfortunately, the favorable effect was only observed in long-acting ß-blockers, but not in short-acting agents, such as esmolol. Dapagliflozin, a sodium-glucose transporter 2 inhibitor, is a drug of choice for treating heart failure with preserved ejection fraction [7]. Administering them orally before and after surgery may improve renal oxygen supply in patients undergoing cardiac surgery, preventing the development of AKI. While they clearly benefit renal circulation, they pose a risk to the kidneys due to an increased incidence of urinary tract infections.

### Renal microcirculation

Recently, the beam of light has been directed onto renal microcirculation. Wang et al. conducted a bibliometric analysis of publications on this topic from 1990 to 2023 [14]. A total of 7,462 records were found in Web of Science, including 6,086 articles and 1,376 reviews, with an annual peak of around 500 publications in 2021. These publications originated from 5,669 institutions across 105 countries and regions and were disseminated through 1,582 journals. The top three journals were the American Journal of Physiology-Renal Physiology (261 articles), Kidney International (191 articles), and the Journal of the American Society of Nephrology (122 articles). The United States was the leading country (34.8% of the global output, with contributions from institutions such as the Mayo Clinic and Harvard University), followed by China (11%), Germany (8.8%), and England (7.5%).

Zhi et al. conducted a prospective observational study in critically ill patients to explore the performance of the renal resistive index (RRI), semiquantitative power Doppler ultrasound (PDU) score and renal venous Doppler waveform (RVDW) pattern in predicting AKI [15]. Of the patients, 31.6% had sepsis, 36.8% had acute heart failure, 5.6% had polytrauma, 6.0% had acute exacerbations of chronic obstructive pulmonary disease, 3.0% had liver failure, and 17.1% had other causes. Measurements were performed within 24 h after admission, provided that a mean arterial pressure (MAP) of at ≥65 mmHg had been achieved with fluid therapy or vasoactive drugs. Although RRI was independently associated with stage 3 AKI, incorporating it into a clinical model (including serum creatinine level on admission, serum lactate level, and urine volume) did not improve predictive performance. It is important to note that the RRI should not be interpreted solely in terms of renal intraparenchymal vascular resistance. The RRI is significantly influenced by systemic haemodynamic factors, particularly by systemic arterial compliance (i.e., increased arterial stiffness) and pulse pressure. These factors also affect renal microcirculation and Doppler-method related parameters. Multivariate binary logistic regression revealed that lactate level, urine volume, serum creatinine level at admission, PDU score, and RVDW pattern predicted stage 3 AKI within 5 days. The authors combined these five variables into Model 1. Model 2 showed the best predictive performance during ROC analysis when RRI was added to Model 1.

The vast majority of publications study global renal oxygenation parameters, which mainly reflect cortical oxygenation. However, the lowest tissue oxygen tension is observed at the tip of the pyramids; therefore, the medulla is the most vulnerable part of the kidney to a septic insult, for instance. Intermittent or continuous measurement of urinary pO_2_ has emerged as a possible clinical tool for monitoring medullary hypoxia. However, its reliability is not widely accepted due to multiple theoretical and technical obstacles. Ji et al. aimed to overcome these difficulties by measuring the time-weighted average puO_2_ – determined by summing the mean puO_2_ values at consecutive time points, multiplying by the time between them, and dividing by the total time – in 76 consecutive adult patients with sepsis and critical illness [16]. They found that puO_2TW_ below 68 mmHg was associated with a higher incidence of septic AKI (positive predictive value: 55.6%, negative predictive value: 86.0%, odds ratio (OR): 7.00, *p* = 0.007). These results highlight the importance of monitoring microcirculation in research.

## Future directions

Arterial stiffness can be measured non-invasively using carotid-femoral pulse wave velocity (cf-PWV) and contributes to acute and chronic renal failure associated with heart failure. The first results of the ongoing study are expected to be published soon [17].

To further clarify the clinical picture:

- Prospective studies are required to validate the experimental and retrospective data.
- The investigation period should be longer than 24 hours, ideally one week or more.
- One size does not fit all: the optimal MAP can differ between patients with or without hypertension, in those receiving angiotensin-converting enzyme inhibitor/receptor blocker users earlier, those with end-stage liver disease, among others.
- Postoperative, cardiac, septic and cardiothoracic surgery patients should be studied separately.
- Not only MAP, but also other pressures (venous, intra-abdominal and lymphatic) and microcirculation should be taken into account.
- Developing an effective prevention algorithms should be of highest priority.

## Conclusion

Understanding renal hemodynamics leads to time- and cost-effective patient care. Several elements of monitoring system are already available in most hospitals and clinicians should seek vigorous implementation of existing knowledge in daily practice. Further research can help to extend our knowledge and refine guidelines.

## Data Availability

No new data were generated or analyzed in this editorial.
